# The Importance of Being “That” Colorectal pT1: A Combined Clinico-Pathological Predictive Score to Improve Nodal Risk Stratification

**DOI:** 10.3389/fmed.2022.837876

**Published:** 2022-02-14

**Authors:** Alessandro Gambella, Enrico Costantino Falco, Giacomo Benazzo, Simona Osella-Abate, Rebecca Senetta, Isabella Castellano, Luca Bertero, Paola Cassoni

**Affiliations:** ^1^Pathology Unit, Department of Medical Sciences, University of Turin, Turin, Italy; ^2^Molecular Pathology Unit, “Città della Salute e della Scienza di Torino” University Hospital, Turin, Italy; ^3^Pathology Unit, Department of Oncology, University of Turin, Turin, Italy

**Keywords:** colorectal carcinoma, pT1, lymph node metastasis, predictive score, age at diagnosis, tumor budding, lymphovascular invasion, tumor-infiltrating lymphocytes

## Abstract

The management of endoscopically resected pT1 colorectal cancer (CRC) relies on nodal metastasis risk estimation based on the assessment of specific histopathological features. Avoiding the overtreatment of metastasis-free patients represents a crucial unmet clinical need. By analyzing a consecutive series of 207 pT1 CRCs treated with colectomy and lymphadenectomy, this study aimed to develop a novel clinicopathological score to improve pT1 CRC metastasis prediction. First, we established the clinicopathological profile of metastatic cases: lymphovascular invasion (OR: 23.8; CI: 5.12–110.9) and high-grade tumor budding (OR: 5.21; CI: 1.60–16.8) correlated with an increased risk of nodal metastasis, while age at diagnosis >65 years (OR: 0.26; CI: 0.09–0.71) and high tumor-infiltrating lymphocytes (OR: 0.19; CI: 0.06–0.59) showed a protective effect. Combining these features, we built a five-tier risk score that, applied to our series, identified cases with a higher risk (score ≥ 2) of nodal metastasis (OR: 7.7; CI: 2.4–24.4). Notably, a score of 0 was only assigned to cases with no metastases (13/13 cases) and all the score 4 samples (2/2 cases) showed nodal metastases. In conclusion, we developed an effectively combined score to assess pT1 CRC nodal metastasis risk. We believe that its adoption within a multidisciplinary pT1 unit could improve patients' clinical management and limit surgical overtreatment.

## Introduction

Screening programs improved the early detection of colorectal cancers (CRCs) (1–5) but raised a new clinical issue: the management of endoscopically resected pT1 colorectal tumors (pT1 CRC).

According to the Tumor Node Metastasis (TNM) classification of malignant tumors, pT1 CRC is defined by the presence of submucosal invasion and represents the earliest stage with metastatic potential (6). Specifically, nodal metastasis in this setting has been reported in up to 15% of pT1 CRC cases (7, 8).

Following pT1 CRC endoscopic resection, two different approaches can be undertaken: either conservative endoscopic follow-up (9, 10) or surgical resection enabling nodal status assessment (11–13). This choice is guided by the risk of node metastasis which is estimated according to specific histopathological parameters which stratify these tumors in low- and high-risk pT1 CRC (14, 15). The ultimate clinical aim is to balance staging/therapeutic benefits with surgical risks and consequences (16, 17).

Regarding the analyzed histopathological features, tumor budding, lymphovascular invasion, tumor grading, and micro staging repeatedly resulted to be significantly associated with nodal involvement: currently, in the presence of even one of these morphological features, surgical resection is proposed, thus favoring lymph node metastases identification but also increasing the percentages of cases submitted to surgery with no lymph node involvement (7, 18).

Based on this observation, a significant subgroup of patients is overtreated (19, 20). In these cases, colectomy with lymphadenectomy is stadiative rather than curative, and it exposes patients to surgery-related morbidity and mortality risks that could be avoided through improved patient selection. Improvement of the risk estimation of nodal metastasis in this setting is an unmet clinical need and would help avoid these risks. In particular, it has to be considered that most of the patients without evidence of node metastasis after surgery were surgically treated because of the presence of a single high-risk parameter, thus novel approaches should focus on developing combined multiparametric scores (7, 8, 18, 21).

Fostered by this state of the art, this study aims to improve pT1 CRC risk stratification by proposing a novel and effective clinicopathological predictor score of nodal metastases, established through the analysis of a comprehensive range of clinical and histopathological features in a large consecutive series of pT1 CRC, surgically treated at a tertiary referral institution.

## Materials and Methods

### Dataset Construction

This is a retrospective study based on a consecutive series of surgically resected pT1 CRC samples (colectomy plus lymphadenectomy) collected from the pathology archives of the “Città della Salute e della Scienza” University Hospital in Turin and diagnosed from January 2010 to March 2019. Demographic, clinical, and histopathological data were extracted from the diagnostic reports, and then pseudo-anonymized by a Unit member not involved in the study. Cases receiving neoadjuvant treatment (*i.e.*, chemoradiotherapy) or lacking material for histological revision were excluded. A final series of 207 samples were collected.

**Figure 1:**
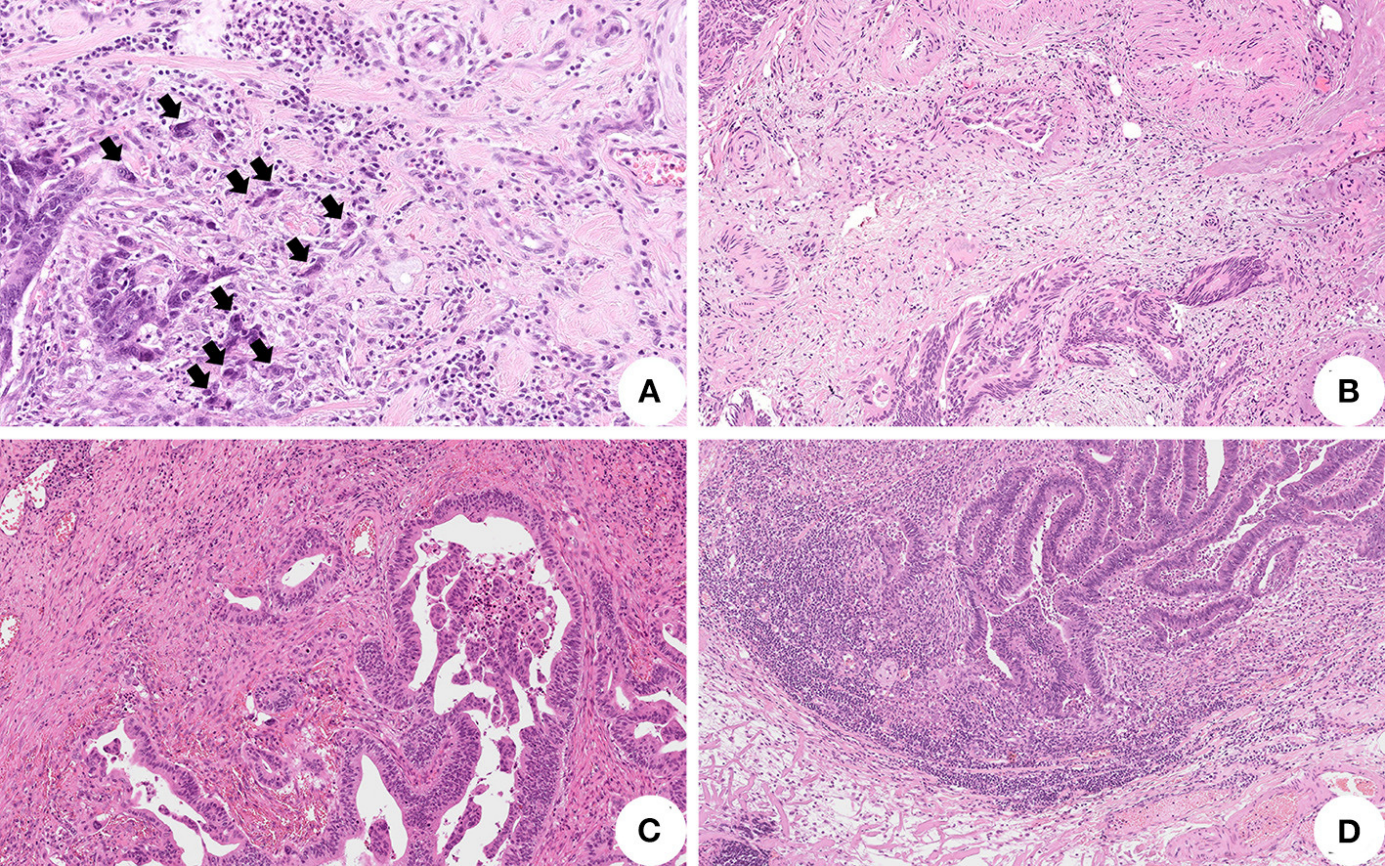
Histopathological features associated with lymph node metastasis in pT1 colorectal cancer (CRC). **(A)** High-grade tumor budding represented by equal or more than five clusters of less than five tumor cells at the invasive front of the tumor (black arrows) (original magnification: 200X); **(B)** Tumor cells invasion of peritumoral stromal vessels representing lymphovascular invasion (original magnification: 100X); **(C)** Absent/mild tumor-infiltrating lymphocytes surrounding tumor cells (original magnification: 100X); **(D)** High tumor-infiltrating lymphocytes facing the invasive tumor front (original magnification: 100X).

Our study was based on the assessment of hematoxylin-eosin (H&E) slides only as per routine practice at our institution. Original H&E slides were retrieved and then independently reviewed by three pathologists (AG, LB, and PC). Disagreements were discussed and jointly resolved by consensus. Histopathological features were assessed and scored according to published studies and international guidelines (Figure 1).

In particular, a tumor “bud” was defined as a cluster of less than five cells located at the invasive front of the tumor (22–24). Based on the clusters quantity, tumor budding was further stratified into a low (less than five clusters per hotspot in a field measuring 0.785 mm^2^) and high grade (equal or more than five clusters per hotspot in a field measuring 0.785 mm^2^) according to a binary system as reported in the literature (8, 22, 25, 26).

Lymphovascular invasion was reported whether tumor cells were identified within endothelial-outlined peritumoral stromal vessels (24). Tumor differentiation grade was attributed according to the least differentiated tumoral component (24). The depth and width of submucosal invasion were assessed and analyzed using 1 and 4 mm cutoff values, respectively (27–29).

**Figure 2:**
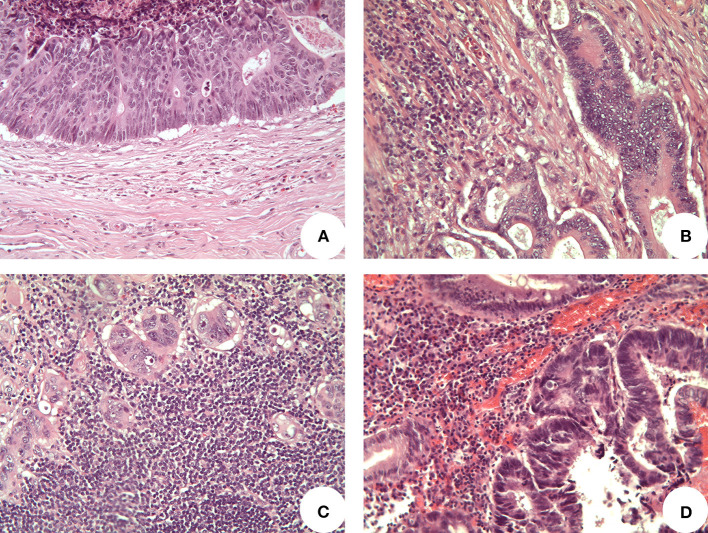
Representative images of tumor infiltrating lymphocytes (TILs) (original magnification 200x). **(A)** “Absent TILs” cases presented no lymphocytes at the invasive front of tumor; **(B)** “mild TILs” cases showed scattering lymphocytes partially surrounding tumor cells; **(C)** “high TILs” cases presented several lymphocytes continuously outlining tumor cells at the invasive tumor front and extending in the surrounding stroma; **(D)** lymphocytes located in superficial tumor layers were not considered, as well as lymphocytes within the tumor bulk.

Regarding the tumor-infiltrating lymphocytes (TILs) assessment, standardized international definition criteria are currently lacking, particularly in the pT1 CRC setting. Therefore, we graded TILs with a semi-quantitative three-tier approach, evaluating the quantity and location of lymphocytes. In particular, we considered only TILs outlining tumor cells at the invasive tumor front, whereas TILs/lymphocytes interspersed within the tumor bulk and/or located in superficial tumor layers were neglected to avoid potential confounders related to luminal/mechanic injury and tumor superficial erosion/necrosis. Then, cases with no peritumoral lymphocytes were reported as “absent TILs,” whereas “high TILs” were cases with lymphocytes continuously outlining tumor cells at the tumor invasive front and extending in the Surrounding stroma. “Mild TILs” referred to all the cases with intermediate features (interrupted outlining and/or infiltrate limited to tumor cells surrounding area) (Figure 2).

**Figure 3:**
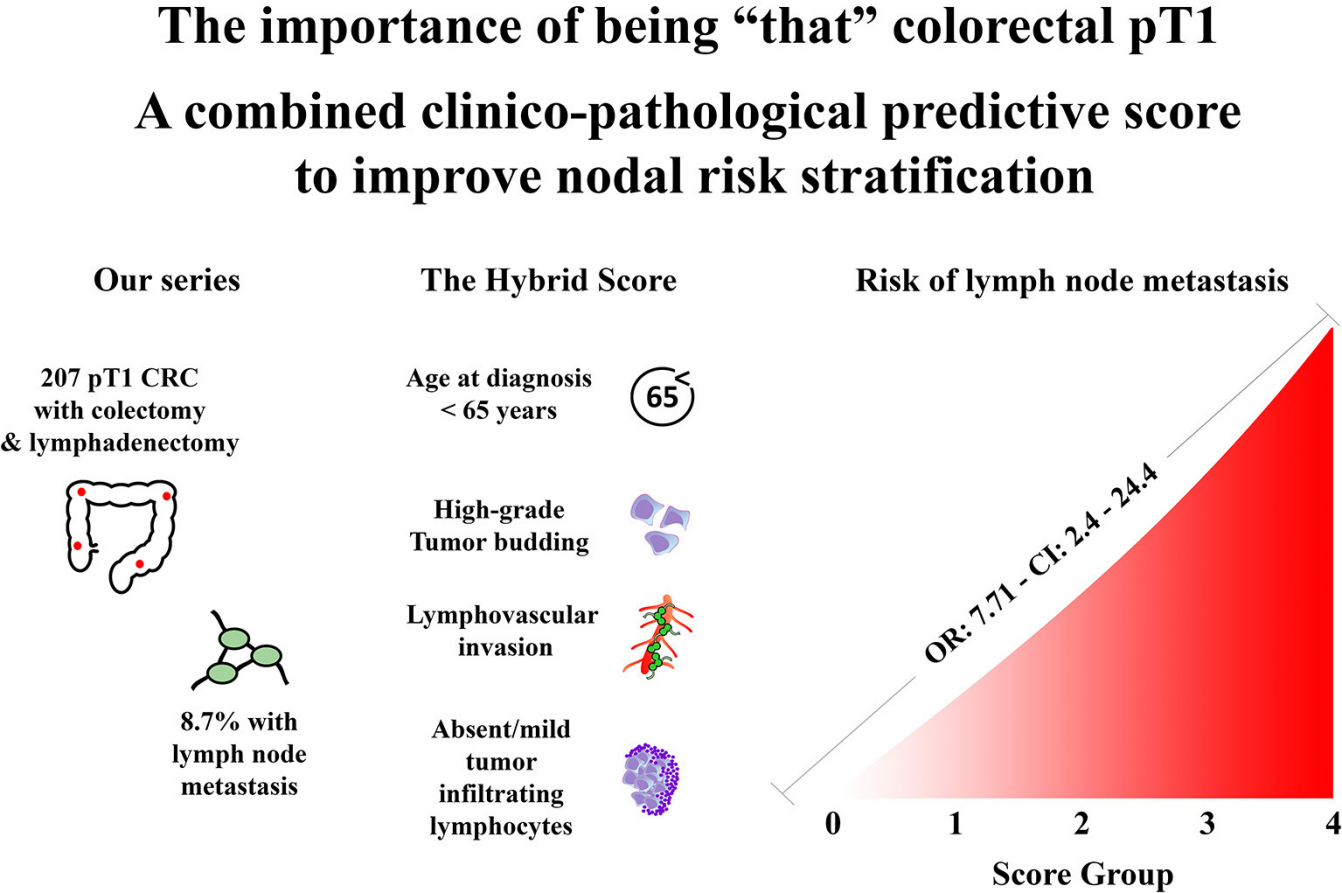
Graphical representation of our study.

The clinical and histopathological features evaluated in this study are reported in Table 1, whereas the overall study design is represented in Figure 3.

**Table 1:** Clinical and histopathological variables evaluated in our study.

**Characteristic**		**Total (*n* = 207)**	**Percentage (%)**
Gender	Male	93	44.9
	Female	114	55.1
Age	Median (interval)	70 (37–90)	–
Age	<65	66	31.9
	≥65	141	68.1
Morphology	Non pedunculated	177	85.5
	Pedunculated	30	14.5
Site	Cecum-Right	69	33.3
	Transverse	14	6.8
	Left	11	5.3
	Sigmoid-rectum	113	54.6
	Sigmoid	67	32.4
	Rectum	46	22.2
Simultaneous polyps	Absent	165	79.7
	Present	42	20.3
Grade	Well differentiated (G1)	31	15.0
	Moderately differentiated (G2)	169	81.6
	Poorly differentiated (G3)	7	3.4
Mucinous feature	Absent	173	83.6
	Present	34	16.4
Lymphovascular	Absent	199	96.1
invasion	Present	8	3.9
TILs	Absent	24	11.6
	Mild	165	79.7
	High	18	8.7
Tumor budding	Low-grade	189	91.3
	High-grade	18	8.7
Sampled lymph nodes	<12	88	42.5
	≥12	119	57.5
Lymph nodes	Absent	189	91.3
metastasis	Present	18	8.7
Growth pattern	Expansive	73	35.3
	Infiltrative	134	64.7
Depth of invasion	<1 mm	22	10.6
	≥1 mm	185	89.4
Width of invasion	<4 mm	44	21.3
	≥4 mm	163	78.7

### Statistical Analysis

Statistical analyses were performed with the Stata 15.0 statistical software (StataCorp, College Station, TX, USA.), applying proper tests for categorical (Pearson's Chi-squared -χ2- test) and numerical variables (T Student's test, Wilcoxon signed-rank test). Bonferroni correction was performed for multiple groups correlations. Univariate binary logistic regression analyses were performed to assess the correlation between the lymph node metastatic status and other variables, calculating relative Odds Ratio (OR) and 95% CI. Statistical analyses were considered significant according to the standard *p*-value <0.05.

## Results

### Clinical and Histopathological Features

Our population presented a median age of 70 years (interval: 37–90) and was mainly composed of female patients (114 cases; 55.1%). Most of the collected lesions were non-pedunculated polyps (177 cases; 85.5%), sited in the sigmoid-rectal tract (113 cases; 54.6%), and 46 specifically located in the rectum (22.2%), without synchronous polyps elsewhere (165 cases; 79.7%). Most cases presented an infiltrative growth pattern (134 cases; 64.7%) and were graded as moderately differentiated (G2) (169 cases; 81.6%), while mucinous features were frequently absent (173 cases; 83.6%). TILs were mild in most cases (165 cases; 79.7%), while high-grade tumor budding and lymphovascular invasion were observed in a minority (8.7 and 3.9%, respectively) of cases. With regards to the Ueno's method for pT1 micro-staging (28), most of the lesions presented a depth of invasion ≥ 1 mm (185 cases; 89.4%) and a width of invasion ≥ 4 mm (163 cases; 78.7%). All clinical and histopathological data are reported in Table 1.

### Lymph Node Metastasis: Defining “That” pT1 Profile

We analyzed the association between all the assessed variables and lymph node involvement to build our predictive risk score.

We registered 18 cases with lymph node metastasis (8.7%). In this population, we observed that nodal involvement was correlated with younger age (cut-off: 65 years) (*p* = 0.005), presence of lymphovascular invasion (*p* < 0.001), absent/mild TILs (*p* = 0.006), and high-grade tumor budding (*p* = 0.003) (Table 2).

**Table 2:** Distribution of clinical data and pathological features based on lymph nodes metastasis.

			**Lymph node metastasis**		
		**Total**	**No**	**Yes**	***p*-value**
		**(*n* = 207)**	**(*n* = 189)**	**(*n* = 18)**	
Gender	Male	93	106	8	0.343
	Female	114	83	10	
Age	Median (interval)	70 (37–90)	70 (43–90)	62 (37–85)	0.025
Age	<65	66	55	11	0.005
	≥65	141	134	7	
Morphology	Non pedunculated	177	164	13	0.094
	Pedunculated	30	25	5	
Site	Cecum-Right	69	63	6	0.997
	Transverse	14	13	1	
	Left	11	10	1	
	Sigmoid-rectum	113	103	10	
Simultaneous polyps	Absent	165	150	15	0.689
	Present	42	39	3	
Mucinous feature	Absent	34	31	3	0.635
	Present	173	158	15	
Lymphovascular invasion	Absent	199	186	13	<0.001
	Present	8	3	5	
TILs	Absent	24	18	6	0.006
	Mild	165	153	12	
	High	18	18	0	
Tumor budding	Low-grade	189	176	13	0.003
	High-grade	18	13	5	
Sampled lymphnodes	<12	88	79	9	0.236
	≥12	119	110	9	
Growth pattern	Expansive	73	70	3	0.084
	Infiltrative	134	119	15	
Depth of invasion	<1 mm	22	20	2	0.945
	≥1 mm	185	169	16	
Width of invasion	<4 mm	44	42	2	0.271
	≥4 mm	163	147	16	

The univariate logistic regression confirmed these data, revealing that lymphovascular invasion (OR: 23.8; CI: 5.12–110.9) and high-grade tumor budding (OR: 5.21; CI: 1.60–16.8) were significantly associated with an increased risk of nodal metastasis, while age at diagnosis >65 years (OR: 0.26; CI: 0.09–0.71) and high TILs (OR: 0.19; CI: 0.06–0.59) showed a protective effect (Table 3).

**Table 3:** Univariate logistic regression analysis between clinico-pathological variables and lymph nodes metastasis.

	**OR**	**CI**	***p*-value**
Gender (Male vs. Female)	1.59	0.60–4.22	0.346
Age (linear)	0.93	0.88–0.97	0.003
Age (>65-year-old)	0.26	0.09–0.71	0.008
Lymphovascular invasion	23.8	5.12–110.9	<0.001
High-grade tumor budding	5.21	1.60–16.8	0.006
High TILs	0.19	0.06–0.59	0.004
Pedunculated morphology	2.52	0.82–7.68	0.103
Grading	2.28	0.63–8.29	0.210
Sampled lymph nodes >12	0.72	0.27–1.89	0.503
Sigmoid-rectum site	1.04	0.39–2.76	0.931

Fostered by the increasing evidence supporting both tumor budding relevance in pT1 CRC risk assessment and its relationship with lymphovascular invasion, we decided to stratify our population according to this parameter to verify its significance in our series. We compared samples with low-grade (189 cases; 91.3%) and high-grade (18 cases; 8.7%) tumor budding observing that high-grade tumor budding strongly correlated with lymph node metastasis (*p* = 0.003) and with lymphovascular invasion (*p* < 0.001), as well. In addition, the univariate logistic regression confirmed that lymphovascular invasion (OR: 13.21; CI: 2.98–58.55) was significantly correlated with high-grade tumor budding.

Finally, we also analyzed the role of tumor grade, another variable routinely used to predict lymph nodes metastasis (7), but we did not identify any correlation with lymph node metastasis (*p* = 0.456).

Since our consecutive series included also 88 cases with <12 sampled lymph nodes and considering the higher risk of recurrence and understanding associated with this sampling, we decided to perform the same analysis excluding non-metastatic cases (N0) with <12 lymph nodes (n = 79) and performed our analysis in the resulting series [thus composed of metastatic cases (N+) and non-metastatic cases (N0) with > 12 lymph nodes (n = 128)]. In this subgroup, we observed the same significant correlations between nodal involvement and clinical-pathologic variables [younger age (*p* = 0.006), presence of lymphovascular invasion (*p* < 0.001), absent/mild TILs (*p* = 0.006), and high-grade tumor budding (*p* = 0.008)], confirmed by univariate logistic regression [lymphovascular invasion (OR: 20.7; CI: 3.65–118.1) and high-grade tumor budding (OR: 4.90; CI: 1.39–17.2) associated with an increased risk of nodal metastasis; age at diagnosis >65 years (OR: 0.24; CI: 0.09–0.70) and high TILs (OR: 0.16; CI: 0.05–0.49) associated with a protective effect].

### Prediction of Lymph Node Metastasis: Assembling the Combined Score

Based on these results, we combined the features significantly related to lymph node metastasis (i.e., age at diagnosis, tumor budding, lymphovascular invasion, and TILs) to build our clinico-histopathological metastatic risk score.

We conferred at one point that any of the following features were present: age at diagnosis <65-year, high-grade tumor budding, presence of lymphovascular invasion, and absent/mild TILs. Therefore, the score would define five possible subgroups ranging from Score 0 (no variables) to Score 4 (all variables simultaneously present).

Applying the score to the whole population, scores 1 and 2 were the most represented groups (121 cases and 63 cases, respectively). Nodal metastasis significantly correlated with our score stratification (*p* < 0.001) and, of note, no metastatic cases were observed in the score 0 groups (n = 13), whereas all score 4 cases (n = 2) presented lymph node metastasis (Table 4).

**Table 4:** Correlation analysis between the score group and presence of lymph node metastasis.

**Score group**	**Lymph node metastasis**		***p*-value**
	**No**	**Yes**	
0	13	0	<0.001
1	117	4	
2	54	9	
3	5	3	
4	0	2	

In addition, the correlation (*p* < 0.001) was maintained even when comparing cases with a score ≥2 and cases with a score of 0 or 1 (Table 5).

**Table 5:** Correlation analysis clustering cases with score <2 vs. score ≥2.

**Score group**	**Lymph node metastasis**		***p*-value**
	**No**	**Yes**	
0–1	130	4	<0.001
2–4	59	14	

Univariate logistic regression also confirmed the correlation between these two clusters: score ≥2 cases presented a significantly higher risk of nodal metastasis compared to score 0 and 1 cases (OR: 7.71; CI: 2.4–24.4) (Table 6).

**Table 6:** Univariate logistic regression evaluating our score outcomes predicting lymph node metastasis.

		**OR**	**CI**	***p*-value**
Score group	Score 2–4 vs. score 0–1	7.71	2.4–24.4	0.001
(clustered score)				

These analyses were also performed in the previously described subgroup [N+ and N0 with >12 lymph nodes (n = 128)], showing similar outcomes either considering the scoring groups separately (Table 7) or clustered in score 0/1 vs. score ≥2 (Table 7), the latter confirmed by univariate logistic regression (Table 8).

**Table 7:** Correlation analysis between the score groups and presence of lymph nodes metastasis in the N+ plus N0 with >12 lymph nodes subgroup.

**Score group**	**Lymph node metastasis**		***p*-value**
	**No**	**Yes**	
0	7	0	<0.001
1	71	4	
2	29	9	
3	3	3	
4	0	2	
0–1	78	4	<0.001
2–4	32	14	

**Table 8:** Univariate logistic regression evaluating the association between the score groups and lymph node metastasis in the N+ plus N0 with >12 lymph nodes cases.

		**OR**	**CI**	***p*-value**
Score group	Score 2–4 vs. score 0–1	8,2	2.5–26.7	0.001
(clustered score)				

## Discussion

This study aimed to enhance the clinical management of endoscopically resected pT1 CRC by identifying and combining metastasis-correlated clinicopathological features in a predictive score of nodal metastasis.

Our series of retrospective consecutive pT1 CRC presented a percentage of nodal metastasis (8,7%) in agreement with the literature range of 4.9–16.9% (7, 8, 19, 21). This data shows that, to date, a large number of cases are potentially overtreated and exposed to significant surgical-related risks without certain benefits except for achieving definitive pathological tumor staging.

Until now, the prediction of CRC pT1 nodal status has been based on histopathological features alone assessed on endoscopically-resected specimens. According to the current international guidelines, the presence of even one single histopathological parameter among poorly differentiated tumors (G3), high-grade tumor budding, lymphovascular invasion, and submucosal invasion ≥1 mm depth or ≥ 4 mm width is routinely used to assign a high risk of node metastasis, aiming for identifying any potential metastatic case but leading to the surgical overtreatment of patients with no metastatic disease (11, 13, 28–33). Of note, these features proved to correlate with node metastasis although a somewhat disappointing interobserver agreement (34, 35). In our study, we confirmed lymphovascular invasion and high-grade tumor budding, and additionally identified absent/mild TILs and younger age (cut-off: 65 years) as relevant features related to nodal involvement and, therefore, we combined them to build our risk score.

Among these, tumor budding and lymphovascular invasion have been thoroughly studied and their association with nodal metastasis is well recognized (8, 18, 33, 36). Recent evidence also suggested that these features are intertwined and could represent two sides of the same coin, ultimately leading to lymph node metastasis (21) since cells composing the tumor “buds” were found to be also responsible for the invasion of nearby stromal lymphovascular vessels. These cells exhibited epithelial and mesenchymal markers suggesting the activation of the epithelial-mesenchymal transition process, an aggressive tumor phenotype associated with poor survival and resistance to therapy (37–41). Our data support their strong reciprocal intertwining and their influence on lymph nodes metastasis development.

Our results also demonstrate that TILs are significantly correlated with the risk of harboring a nodal metastasis, a finding in agreement with the established role of the immune system in influencing CRC growth and survival (42–45). However, it should be noted that so far, studies evaluating the role of TILs in CRC mainly focused on advanced disease settings. The few reports addressing pT1 CRC provided conflicting results. (43, 44, 46–48). A univariate analysis of 102 endoscopically resected pT1 CRCs reported an increased number of CD3 positive TILs in the metastatic group, but this finding was not confirmed by multivariate analysis (21). No correlations were observed analyzing the CD8 positive TILs (21). Differently, a recent study by Kang et al. analyzed the distribution and mean numbers of CD3, CD8, and FOXP3 positive lymphocytes in pT1 CRC and identified a significant association between lymph node metastasis and a lower number of CD8 positive lymphocytes located within the tumor core (49). In the present study, we demonstrated a correlation between absent/mild TILs and a higher risk of nodal involvement. This association seems to be particularly relevant since none of our cases with high TILs presented nodal metastasis, while the four score 1 cases presenting nodal metastasis received this score exactly due to the presence of absent/mild TILs. We believe that TILs could become a significant histopathological feature of pT1 CRC, representing a morphological counterpart of tumor immunobiology and immune system activation against tumor growth. In this regard, our results support the relevance of this parameter in shaping tumor invasiveness even at early disease stages, but further studies should specifically investigate TILs in early CRC to confirm their predictive significance in terms of nodal metastasis and provide additional insights on their mechanistic role and develop specific guidelines regarding their assessment.

Lastly, the predictive significance of age at diagnosis is of particular interest since it shows the importance of clinical variables even in this early oncological setting. Moreover, whereas the assessment of histopathological features alone can be hampered by inter-observer variability (50), age at diagnosis is not. Younger age is also known to be related to more aggressive CRC lesions (51–54).

We did not confirm micro staging (≥1 mm depth and ≥4 mm width of invasion) nor tumor grading as potential risk factors of nodal involvement. Similar evidence has been recently reported in the literature: in particular, micro staging reliability in pT1 CRC has been questioned for the significant influence of technical processing and lesion morphology on its interpretation; similarly, the importance of tumor grading as a predictive marker has been found to be overall limited compared with other morphological features (55–59).

In the practical daily routine, some pT1 CRC cases, although close to the recommended cut-off, did not meet the required number of harvested lymph nodes. Indeed, this is a limitation that actually occurs, potentially impacting the pT1 CRC staging (60, 61). Aiming to develop a score that can be applied in a “real-world” pT1 CRC series, we preferred to also maintain these cases and test our score in both scenarios, thus considering either all cases (including the ones with <12 lymph nodes) or the subgroup of N+ and N0 >12 only cases. Indeed, our score maintains the same level of statistic and clinical significance, further confirming its practical relevance.

We acknowledge that our study presents some potential limitations, including its retrospective nature and the limited, although considerable, sample size. Moreover, future studies should investigate the efficacy of the here proposed approach on endoscopic resection samples.

Ultimately, our score improves the overall identification of patients who would benefit from a surgical approach following endoscopically resected pT1 CRC compared to the evidence reported so far in the literature (62–64). It should also be noted that balancing the risks due to over-and undertreatment is extremely challenging when dealing with an early tumor stage. Identifying subgroups of patients, no matter how small, with very low or very high metastatic risks is particularly important since it allows to confidently propose a therapeutic option to these patients. Our score enabled this result since no score 0 patient showed nodal metastasis, while all score 4 had metastatic disease. Although the sample sizes of these groups were particularly limited, these promising results warrant further validation. Among patients with intermediate-risk scores (score 1–3), we observed a progressive increase of nodal metastasis (score 1: 3.4%, score 2: 16.7%, score 3: 60.0%), a piece of information that would not have been available by employing the conventional dichotomized approach. This stratified risk assessment could help tailor patients' management by multidisciplinary boards.

In conclusion, despite some limitations, including the retrospective and single-institutional nature, our study allowed us to develop a novel multiparametric score combining evidence-based histopathological features with age at diagnosis as a clinical variable. Our score represents an effective system to estimate the lymph node metastasis risk, superior to the current single parameter-based risk assessment. Further multi-centric and ideally prospective studies are recommended to confirm our findings and enable the adoption of the proposed score within multidisciplinary CRC tumor boards to guide patients' management.

## Data Availability Statement

The raw data supporting the conclusions of this article will be made available by the authors, without undue reservation.

## Ethics Statement

The studies involving human participants were reviewed and approved by the Institutional Review Board (or Ethics Committee) of the University of Turin (approval number: DSM-ChBU n° 03/2020, approved in October 2020). Written informed consent for participation was not required for this study in accordance with the national legislation and the institutional requirements.

## Author Contributions

LB and PC: conceptualization and funding acquisition. AG, EF, GB, SO-A, RS, IC, LB, and PC: methodology and writing—review and editing. AG, EF, GB, SO-A, and LB: formal analysis. AG and EF: writing—original draft preparation. All authors contributed to the article and approved the submitted version.

## Funding

This research was funded by the Rete Oncologica del Piemonte e della Valle d'Aosta (Oncology Network of Piedmont and Aosta Valley-Italy; Grant to LB and PC, no specific grant number available).

## Conflict of Interest

The authors declare that the research was conducted in the absence of any commercial or financial relationships that could be construed as a potential conflict of interest.

## Publisher's Note

All claims expressed in this article are solely those of the authors and do not necessarily represent those of their affiliated organizations, or those of the publisher, the editors and the reviewers. Any product that may be evaluated in this article, or claim that may be made by its manufacturer, is not guaranteed or endorsed by the publisher.
